# Protein kinase D1 regulates matrix metalloproteinase expression and inhibits breast cancer cell invasion

**DOI:** 10.1186/bcr2232

**Published:** 2009-02-25

**Authors:** Tim Eiseler, Heike Döppler, Irene K Yan, Steve Goodison, Peter Storz

**Affiliations:** 1 Department of Cancer Biology, Mayo Clinic Comprehensive Cancer Center, Griffin Building, 4500 San Pablo Road, Jacksonville, FL 32224, USA; 2 Department of Surgery, University of Florida, 653 West 8th Street, Jacksonville, FL 32209, USA; 3 Mayo Clinic, Griffin Room 306, 4500 San Pablo Road, Jacksonville, FL 32224, USA

## Abstract

**Introduction:**

The biological and molecular events that regulate the invasiveness of breast tumour cells need to be further revealed to develop effective therapies that stop breast cancer from expanding and metastasising.

**Methods:**

Human tissue samples of invasive breast cancer and normal breast, as well as breast cancer cell lines, were evaluated for protein kinase D (PKD) expression, to test if altered expression could serve as a marker for invasive breast cancer. We further utilised specific PKD1-shRNA and a system to inducibly-express PKD1 to analyse the role of PKD1 in the invasive behaviour of breast cancer cell lines in two-dimensional (2D) and three-dimensional (3D) culture. Invasive behaviour in breast cancer cell lines has been linked to matrix metalloproteinases (MMPs), so we also determined if PKD1 regulates the expression and activity of these enzymes.

**Results:**

We found that the serine/threonine kinase, PKD1, is highly expressed in ductal epithelial cells of normal human breast tissue, but is reduced in its expression in more than 95% of all analysed samples of human invasive breast tumours. Additionally, PKD1 is not expressed in highly invasive breast cancer cell lines, whereas non-invasive or very low-invasive breast cancer cell lines express PKD1. Our results further implicate that in MDA-MB-231 cells PKD1 expression is blocked by epigenetic silencing via DNA methylation. The re-expression of constitutively-active PKD1 in MDA-MB-231 cells drastically reduced their ability to invade in 2D and 3D cell culture. Moreover, MCF-7 cells acquired the ability to invade in 2D and 3D cell culture when PKD1 expression was knocked-down by shRNA. PKD1 also regulated the expression of breast cancer cell MMPs, MMP-2, MMP-7, MMP-9, MMP-10, MMP-11, MMP-13, MMP-14 and MMP-15, providing a potential mechanism for PKD1 mediation of the invasive phenotype.

**Conclusions:**

Our results identify decreased expression of the PKD1 as a marker for invasive breast cancer. They further suggest that the loss of PKD1 expression increases the malignant potential of breast cancer cells. This may be due to the function of PKD1 as a negative regulator of MMP expression. Our data suggest re-expression of PKD1 as a potential therapeutic strategy.

## Introduction

Protein kinase D (PKD) belongs to the calcium/calmodulin-regulated kinase family of serine/threonine kinases [1]. The PKD family consists of three members, PKD1/PKCμ, PKD2 and PKD3/PKCν, which share a unique molecular architecture [2]. Depending on the cancer cell type and the activation mechanism, recent reports have revealed important functions for PKD enzymes in the regulation of cell adhesion, vesicle transport and cell survival [3-8]. There is also increasing evidence that PKD enzymes are involved in pathways that inhibit apoptosis in tumours of the pancreas and cervix [5,8].

A potential mechanism for PKD1 regulation of cell survival is via activation of the anti-apoptotic transcription factor nuclear factor (NF) κB [8,9]. PKD1 was also recently implicated in the inhibition of cell migration of pancreatic cancer cells [10]. In line with its negative regulatory effects on cell motility, PKD1 can be activated by the RhoGTPase RhoA [11], which in its active state has also been implicated in the inhibition of cell migration [12,13]. PKD1 expression is downregulated in androgen-independent prostate cancer [14] and the PKD1 promoter is epigenetically-silenced by methylation events in gastric cancer [15]. To date, there was little known on the expression and function of PKD1 in breast cancer. Breast cancer cells invade surrounding tissues by breaking through the basal membrane using invadopodia, which participate in proteolytic matrix degradation. In some breast cancer cells, PKD forms a complex with cortactin and paxillin, which are both associated with invadopodia membranes [4]. However, the function and the activation status of PKD1 in this complex are not known.

An important step in the complex regulation of tumour expansion and metastasis is the degradation of extracellular matrix (ECM) which allows cells to migrate and invade surrounding areas and into peripheral tissues or enter the bloodstream [16,17]. Matrix metalloproteinases (MMPs) are collagenases (e.g. MMP-1, MMP-13), stromelysins (e.g. MMP-10, MMP-12), gelatinases (e.g. MMP-2, MMP-9) or membrane-type enzymes (e.g. MMP-14, MMP-16) and have been recognised as important mediators of ECM degradation. In almost all human cancers the increased expression of certain MMPs correlates with tumour expansion, increased invasiveness and poor prognosis [16,18]. MMPs and MMP inhibitors have been extensively investigated in human breast cancer clinical studies [19-22].

The tissue levels of at least MMP-1, MMP-2, MMP-9, MMP-11, membrane type (MT) 1-MMP, tissue inhibitors of metalloproteinases (TIMP) 1 and TIMP-2 have been correlated with poor outcome of breast cancer patients [20,23,24]. Furthermore, MMP-1 and MMP-2 have been described as genes that selectively mediate lung metastasis in the MDA-MB-231 xenograft model of breast cancer [25] and are members of a lung metastasis gene signature for human breast cancers [26]. Recent data also show that tumour-derived, rather than stromal fibroblast-derived, MMP-13 correlates with aggressive breast tumour types and is inversely correlated with the overall survival of breast cancer patients [22]. The regulation of MMP expression is complex, involving a multitude of transcription factors and histone deacetylases [27-31]; however, no information is available regarding the negative regulation of MMP genes in mechanisms that reduce ECM degradation.

Here we show that PKD1 expression is decreased in invasive breast cancer tissue and that PKD1 expression is silenced in invasive breast cancer cell lines. The re-expression of active PKD1 in highly invasive breast cancer cells blocks cell invasion and the reduction of PKD1 expression in very low-invasive breast cancer cells increases the invasive ability of these cells. We also identify PKD1 as an inhibitor of the expression of matrix-metalloproteinases, such as MMP-2, MMP-7, MMP-9, MMP-10, MMP-11, MMP-13, MMP-14 and MMP-15, all of which have been implicated in the progression of breast cancer. Our findings show that PKD1 inhibits breast tumour cell invasion and thus may influence tumour cell dissemination and metastasis, the most lethal aspect of breast cancer.

## Materials and methods

### Cell lines, DNA constructs, reagents and antibodies

All cell lines were purchased from the American Type Culture Collection (ATCC, Manassas, VA, USA) and were maintained according to information provided by ATCC. pcDNA3-based expression constructs for HA-tagged wildtype PKD1, kinase-dead PKD1 (PKDinactive, PKD1.K612W) and constitutively-active PKD1 (PKDactive, PKD1.Y463E) have been described previously [8]. The doxycycline-regulated expression system for mammalian cells was from Invitrogen (Carlsbad, CA, USA). MDA-MB-231 cells were first stably transfected with pcDNA6/TR and selected with blasticidin to generate a MDA-MB-231-TR cell line. Constitutively-active PKD1 (PKD1active, PKD1.Y463E) was cloned into pcDNA4/TO-B via BamHI and XhoI sites and verified by DNA sequencing.

To generate stable cell lines which allow doxycycline-dependent inducible expression of constitutively-active PKD1, the pcDNA4/TO-B-PKD1.Y463E construct was stably transfected into the MDA-MB-231-TR cells and selected with Zeocin to generate the MDA-MB-231-TR-PKD1.Y463E cell lines. All transfections were performed with Lipofectamine 2000 from Invitrogen (Carlsbad, CA, USA). MCF-7 cells were infected with PKD1-shRNA lentivirus to generate the MCF-7-PKD1.RNAi cell lines. Clonal selection for stably infected cells was performed with puromycin. The rabbit polyclonal antibody specific for PKD1 was raised against a H2N-MAECQNDSGEMQDP-amide peptide corresponding to amino acids 372–385 in human PKD1 (21 Century Biochemicals, Marlboro, MA). The rabbit polyclonal antibody for PKD2 was from Upstate (Charlottesville, VA, USA), and the rabbit polyclonal antibody for PKD3 was from Bethyl Laboratories (Montgomery, TX, USA). All these antibodies were specific for the respective PKD isoenzyme and were not cross-reactive with other PKD family members (data not shown). Anti-FLAG, anti-HA and anti-Actin were from Sigma (St. Louis, MO, USA). Anti-MMP-9 was from BD Biosciences (San Jose, CA, USA) and anti-MMP-2 from Epitomics (Burlingame, CA, USA). The secondary horseradish peroxidase (HRP) linked anti-mouse or anti-rabbit antibodies were from Roche (Indianapolis, IN, USA). The DNA methyltransferase inhibitor RG108 (2-(1,3-Dioxo-1.3-dihydro-2H-isoindol-2-yl)-3-(1H-indol-3-yl)propionic acid) was from Sigma (St. Louis, MO, USA).

### Tissue samples, immunohistochemistry and statistical analysis

Tissue microarray (TMA) slides containing histologically-confirmed human breast cancer and normal human breast tissue samples were purchased from Imgenex (San Diego, CA, USA). The TMAs were deparaffinised (one hour at 60°C), de-waxed in xylene (five times for four minutes) and gradually re-hydrated with ethanol (100%, 95%, 75%, twice with each concentration for three minutes). The rehydrated TMAs were rinsed in water and subjected to antigen retrieval in citrate buffer (pH 6.0) as described by the manufacturer (DAKO, Carpinteria, CA, USA).

Slides were treated with 3% hydrogen peroxide (five minutes) to reduce endogenous peroxidase activity and washed with PBS containing 0.5% Tween 20. PKD1, PKD2 and PKD3 were detected using specific antibodies at a dilution of 1:2000, 1:1000 and 1:200, respectively, in PBS/Tween and visualised using the Envision Plus Dual Labeled Polymer Kit following the manufacturer's instructions (DAKO, Carpinteria, CA, USA). Images were captured using ImagePro software (Media Cybernetics, Bethesda, MD, USA). The TMAs were scored independently by three different experienced scientists. Uniform pre-established criteria were used.

Immunoreactivity was graded semiquantitatively by considering the intensity of the staining of the ductal cells. A histological score was obtained from each sample, which ranged from 0 (no immunoreaction) to 5 (maximum immunoreactivity as seen in normal ductal tissue). All normals were scored between 4 and 5, with an average of all samples at 4.57. Immunostaining was assessed by considering the percentage of positive cells because the positivity was homogeneous in each sample. Reproducibility of the scoring method between three observers was greater than 90%. In the remaining cases, in which discrepancies had been noted, differences were settled by consensus review of corresponding slides. Statistical analysis (student's *t*-test) was performed with GraphPad Software (GraphPad Software, La Jolla, CA, USA).

### Reverse transcription PCR

Cellular mRNA isolation was performed using RNA-Bee (TEL-TEST, Friendswood, TX, USA) according to the manufacturer's instructions and was transcribed into cDNA using Superscript II (Invitrogen, Carlsbad, CA, USA). For the transcription reaction, 1 μg Oligo dT(18) primer (New England Biolabs, Beverly, MA, USA) and 1 μg RNA were incubated in a total volume of 10 μl water at 70°C for 10 minutes. Then, 5× buffer, 40 U RNAsin (Roche, Mannheim, Germany), 200 μM dNTP (New England Biolabs, Beverly, MA, USA), 10 mM DTT, 300 U Superscript II reverse transcriptase were then added to a total volume of 20 μl. The reaction was carried out at 45°C for 60 minutes and then heat inactivated at 95°C for five minutes. The resulting cDNA pool was subjected to PCR analysis using specific primer sets. Primers for human PKD1 were TTCTCCCACCTCAGGTCATC and TGCCAGAGCACATAACGAAG, PKD2 were CAACCCACACTGCTTTGAGA and CACACAGCTTCACCTGAGGA, and PKD3 were TCATTGACAAACTGCGCTTC and GTACATGATCACGCCCACTG. Primers for human MMPs and TIMPs are described elsewhere [32]. The primers for actin were CCTCGCCTTTGCCGATCC and GGATCTTCATGAGGTAGTCAGTC. Reaction conditions for the PCR reaction were: one minute annealing at 55°C, one minute amplification at 72°C, with 20, 35 and 40 cycles.

### Lentiviral shRNA expression

The Lentiviral shRNA expression system to knock-down PKD1 expression is commercially available from Sigma (SHDNA MISSION^® ^shRNA Plasmid DNA; St. Louis, MO, USA). The chosen sequences for siRNA were specific, as judged by BLASTn searches of the *all GenBank+RefSeq Nucleotides+EMBL+DDBJ+PDB sequences *and the *human subset of GenBank+EMBL+DDBJ sequences*. Sequences are available from Sigma (NM_002742.x-2498s1c1 and NM_002742.x-2978s1c1; St. Louis, MO, USA). The ViraPower Lentiviral Expression System (Invitrogen, Carlsbad, CA, USA) was used for an optimised mix of packaging plasmids which supplies the structural and replication proteins that were required to produce Lentivirus in 293FT cells.

### Cell lysates, immunoprecipitation and immunostaining

Cells were lysed in lysis buffer (50 mM Tris/HCl pH 7.4, 1% TritonX-100, 150 mM sodium chloride (NaCl), 5 mM EDTA) plus Protease Inhibitor Cocktail (Sigma, St. Louis, MO, USA) and either lysates were used for immunoblot analysis or proteins of interest were immunoprecipitated by a one-hour incubation with the respective antibody (2 μg) followed by a 30 minute incubation with protein A/G-agarose (Santa Cruz Biotechnology, Santa Cruz, CA, USA). Immune complexes were washed five times with TBS (50 mM Tris/HCl pH 7.4, 150 mM NaCl), resolved by SDS-PAGE, transferred to nitrocellulose and analysed by immunostaining.

### Transwell assay

Migration and invasion assays were performed as previously described [33] using Transwell chambers. Transwell chambers were coated with standard Matrigel (2 μg/well,) from Fisher (Pittsburgh, PA, USA). For assays with transient transfected cells, cells were co-transfected with the constructs of interest and a β-Gal reporter plasmid (pCS2-(n)β-gal) at a ratio of 5:1 for 24 hours. Inserts of transwell plates were coated, dried over night and re-hydrated for one hour with 40 μl of tissue culture media. Cells were harvested, washed once with media containing 1% BSA, re-suspended in media containing 0.1% BSA (10^6 ^cells/ml) and seeded on the Transwells (10^5 ^cells). NIH-3T3 conditioned medium served as a chemoattractant in the lower chamber. Remaining cells were used to analyse the transfection efficiency and/or the expression of proteins of interest. After 16 hours, cells on top of the transwell insert were removed and cells that had migrated to the lower surface of the filters were fixed in 4% paraformaldehyde and stained with X-Gal staining solution. Cells which stained positive for β-Galactosidase expression were counted. The mean of triplicate assays for each experimental condition was used as percentage relative invasion.

### Multicellular spheroids/3D cell culture assay

Three-dimensional (3D) analysis of morphology was performed as described previously [12]. In brief, cell culture dishes (24-well plates) were precoated with undiluted phenol red-free Matrigel (10 mg/ml). In 200 μl PBS, 10^4 ^cells (per well of a 24-well plate) were suspended and then mixed with 100 μl of cold Matrigel (10 mg/ml). The cell suspension was added dropwise over the bottom layer to cover it. After the cell layer was set complete, culture media was added over the top. Media was changed every two days, without disturbing the cell/matrix layer. Photos were taken after indicated days using a 10× magnification for an overview and 40× to document structure.

### Cell proliferation assays

Cells were seeded at a density of 2500 cells/well in clear bottom black 96-well tissue culture plates. After adhesion overnight, the respective t = 0 plate was washed once with 1 × PBS, tapped dry and then frozen at -80°C. The same procedure was used to process the respective t = 24 hour and t = 48 hour time-point plates. After all plates had been acquired, cell proliferation was measured using a CytoQuant cell proliferation assay kit (Invitrogen, Carlsbad, CA, USA). Cells were lysed with 200 μl 1× cell-lysis buffer with CyQuant GR dye (1:400 dilution) per well. CytoQuant GR fluorescence was measured using a SpectraMAX M5 plate reader (Molecular Devices/MDS, Toronto, Canada) by exiting the dye at 485 nm and reading emission at 538 nm.

### Zymography

Zymography was performed as previously described [34]. Briefly, 48 hours after transfection culture media was harvested. Samples were mixed with 2 × loading buffer (50 mM Tris-HCl pH 6.8, 10% (v/v) glycerol, 1% (w/v) SDS, 0.01% (w/v) bromophenol blue) and resolved on an SDS-PAGE containing 0.12 mg/ml gelatin (porcine skin type A, bloom 300). Gels were soaked for one hour in 2.5% Triton X-100, then washed twice with collagenase buffer (50 mM Tris-HCl pH 7.6, 0.2 M NaCl, 5 mM calcium chloride, 0.2% Brij-35) and incubated at 37°C for 18 hours. Gels were then washed with distilled water and incubated in Coomassie brilliant blue staining solution (40% methanol, 10% acetic acid/0.025% Coomassie Brilliant Blue R-250) at room temperature for two hours. Gels were then washed for 24 hours in distilled water and scanned using an Agfa Duoscan T1200 Scanner (Agfa-Gevaert, Mortsel, Belgium).

## Results

### PKD1 expression is reduced in invasive ductal carcinoma

**Figure 1:**
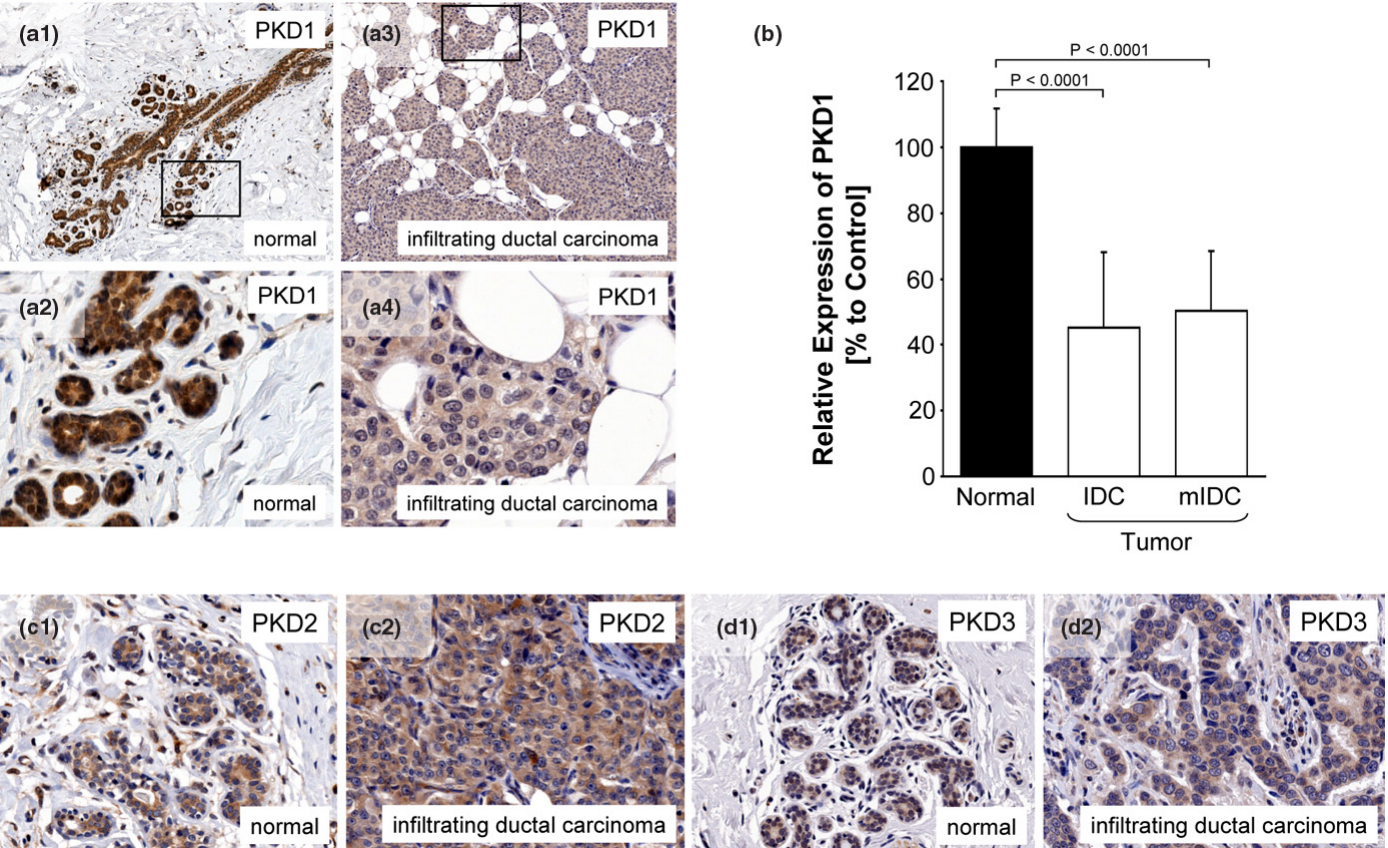
PKD1 expression is reduced in invasive ductal carcinoma. **(a to d) **Tissue microarrays (TMAs) including 10 normal breast tissue samples, 40 invasive ductal carcinoma of the breast and 10 metastatic invasive ductal carcinoma samples from lymph nodes were analysed for the expression of the protein kinase D (PKD) family kinases PKD1, PKD2 and PKD3, using isoform specific antibodies. Representative pictures of normal and tumour tissue are depicted. **(b) **Statistical analysis of the normal and tumour samples. Error bars shown represent standard deviation. P values were acquired with the student's *t*-test using Graph Pad software. P values indicate extreme statistical significance. IDC = invasive ductal carcinoma; mIDC = metastatic invasive ductal carcinoma.

We analysed TMAs including 10 normal breast tissue samples, 40 invasive ductal carcinoma of the breast and 10 metastatic invasive ductal carcinoma samples from lymph nodes for the expression of the PKD family kinases, PKD1, PKD2 and PKD3. We found that PKD1 is highly expressed in epithelial ductal tissue of human normal breast samples, but is reduced in its expression in more than 95% of invasive human breast tumour samples (representative pictures of normal and tumour tissue are in Figures 1a1 to 1a4). When compared with normal breast tissue the tumour samples revealed an approximate 60% reduction in PKD1 expression in both, invasive ductal carcinoma and metastatic invasive ductal carcinoma (Figure 1b). PKD1 may have functions in both the cytosol and the nucleus [35]. PKD1 staining was observed in normal breast tissue in the nuclei, as well as in the cytosol. Breast cancer samples of invasive ductal carcinoma and metastatic invasive ductal carcinoma showed both a decrease of cytosolic staining and nuclear staining. Interestingly, the two other PKD family members, PKD2 and PKD3, showed no significant difference in their expression or localisation in infiltrating ductal carcinoma and normal breast tissue (Figures 1c1 to 1d2), indicating a potential function for PKD1 in invasive breast cancer. For all samples, sex, age, diagnosis, pathological tumour-node-metastasis (pTNM), stage, lymph node (positive lymph nodes/examined lymph nodes) as well as progesterone receptor (PR) and oestrogen receptor (ER) expression status were available. In all 50 samples of invasive ductal cancer we observed a significant reduction of PKD1 expression as compared with the normal ductal tissue – regardless of stage, ER, PR or other above markers. A similar downregulation of PKD1 was also recently described for other cancers such as prostate [14] and gastric cancer [15].

### PKD1 is not expressed in invasive breast cancer cell lines

**Figure 2:**
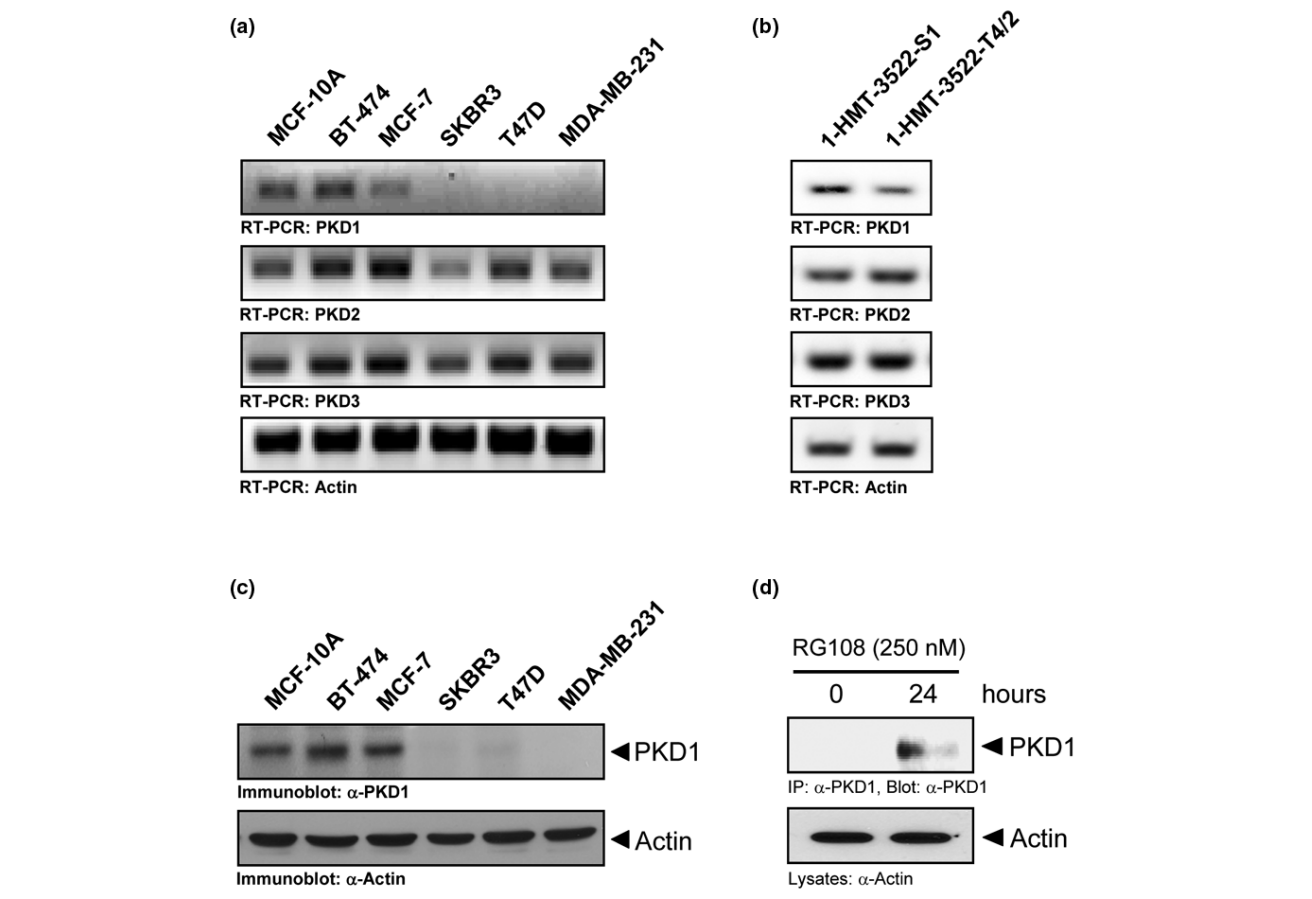
PKD1 is not expressed in invasive breast cancer cell lines. **(a,b) **MCF-10A cells and BT-474, MCF-7, SKBR3, T47D, MDA-MB-231, 1-HMT-3522-S1 or 1-HMT-3522-T4/2 breast cancer cell lines were cultivated under normal growth conditions. mRNA was isolated and the expression of protein kinase D (PKD) 1, PKD2, PKD3 and actin was detected by RT-PCR. **(c)** MCF-10A cells and BT-474, MCF-7, SKBR3, T47D, MDA-MB-231, 1-HMT-3522-S1 or 1-HMT-3522-T4/2 breast cancer cell lines were cultivated under normal growth conditions. Cells were lysed and lysates were analysed for PKD1 expression by western blotting. Actin served as loading control. **(d)** MDA-MB-231 cells were either left untreated or treated with RG108 (250 nM, for 24 hours). PKD1 was immunoprecipitated (α-PKD1 antibody) and immunoprecipitates were analysed for PKD1 expression. Western blotting of lysates for actin (α-actin antibody) served as a control.

We next determined the PKD1 expression status in a subset of breast cancer cell lines. We found that PKD1 expression at the mRNA level is absent in the highly invasive breast cancer cell lines SKBR3, T47D and MDA-MB-231 (Figure 2a). Non-invasive or very low-invasive breast cancer cell lines such as BT-474 and MCF-7 and a normal breast cell line MCF-10A showed moderate PKD1 expression. No distinct pattern was detectable for PKD2 and PKD3 expression when cells with high invasive potential were compared with cell lines with low invasive capacity (Figure 2a). We also analysed the 1-HMT-3522 cell progression model, in which the subclone S1 retains a more benign phenotype, and the subclone T4/2 has a more invasive character [36]. T4/2 cells showed less PKD1 mRNA expression as compared with S1 cells, whereas PKD2, PKD3 and actin mRNA levels were similar (Figure 2b). These data suggest that PKD1 expression is decreased when cells achieve a more aggressive state.

Further, as expected, loss of PKD1 mRNA correlated with a lack of PKD1 protein expression in highly invasive cells lines (Figure 2c). In gastric cancer it was recently shown that PKD1 is downregulated in its expression by DNA methyltransferases [15]. Epigenetic silencing of genes by DNA methylation is also a common mechanism involved in gene silencing in breast cancer. We found that in MDA-MB-231 cells the PKD1 gene is also epigenetically silenced by DNA methylation, because treatment of these cells with agents that inhibit DNA methyltransferases such as RG108 (Figure 2d) or Decitabine (data not shown) led to the re-expression of PKD1.

### Knockdown of PKD1 increases cell invasion

**Figure 3:**
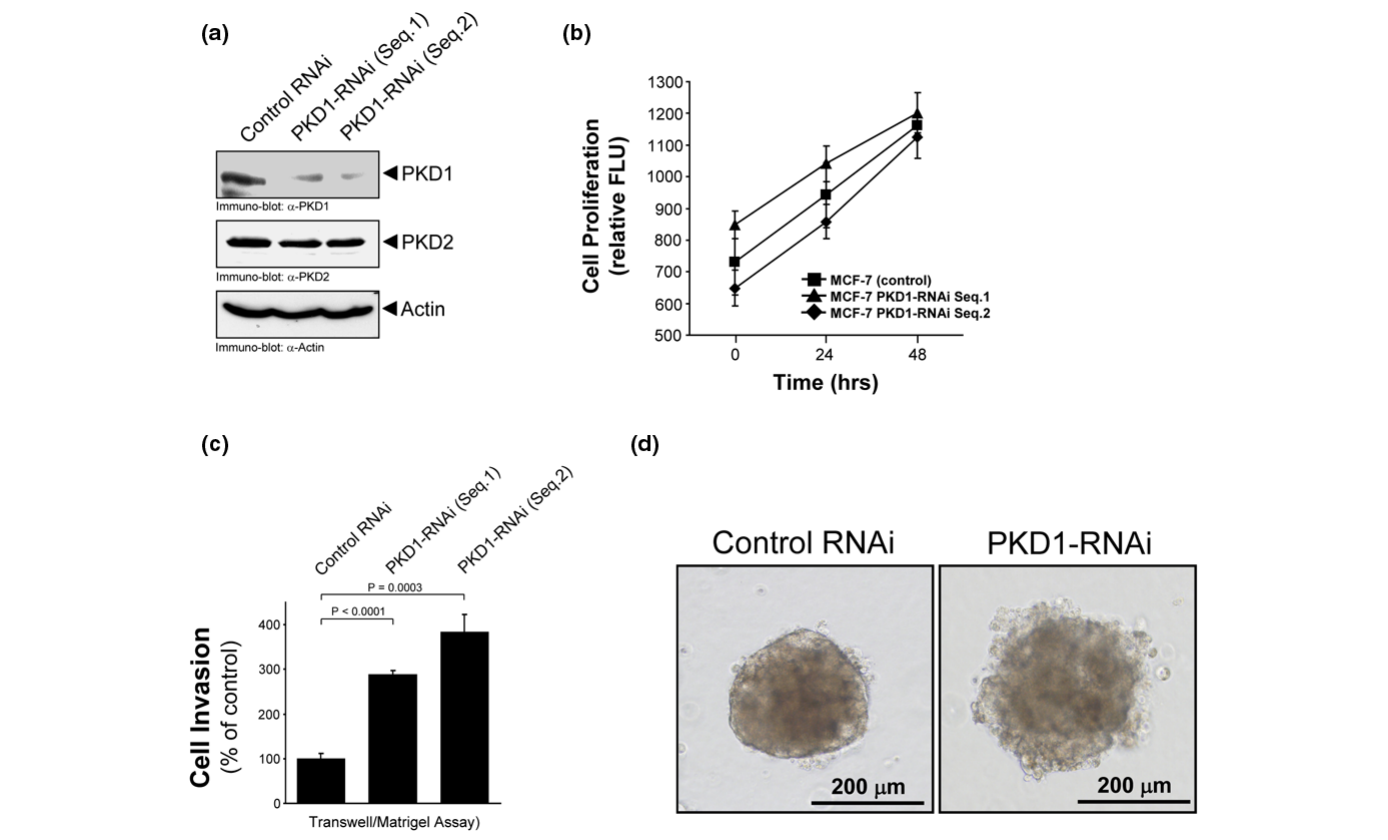
The knockdown of PKD1 increases cell invasion. **(a) **MCF-7 cells were stably transduced with lentivirus coding for two different human shRNA sequences for protein kinase D (PKD) 1 (PKD1-RNAi Seq.1 and Seq.2) or for a non-target sequence which served as control. Cells were lysed and analysed for PKD1 expression by western blotting. Immunostaining for PKD2 and actin expression served as controls. **(b)** MCF-7 control and PKD1-RNAi clones were subjected to a cell proliferation assay using the CyQuant cell proliferation assay kit. **(c) **MCF-7 control and PKD1-RNAi clones were seeded on Matrigel-coated Transwell filters and Transwell invasion assays were performed over a time period of 16 hours. **(d)** MCF-7 cells stably-transfected with control RNAi or PKD1-RNAi were grown in 3D/Matrigel cell culture over a period of 60 days. Cells were photographed at day 60. Bars indicate the size of the spheroids. For experiments shown in b and c, error bars represent standard deviation. P values were acquired with the student's *t*-test using Graph Pad software. All P values indicate statistical significance.

We next analysed if the decreased expression of PKD1 is one of the means by which breast tumour cells may increase their invasive potential. To test this we utilised the very low-invasive breast cancer cell line MCF-7 which we have shown moderately expresses PKD1 (Figures 2a,c). We transfected MCF-7 cells stably with control shRNA or two different PKD1-specific shRNA sequences to knockdown PKD1 expression (Figure 3a). Both shRNA sequences led to an approximate 80% reduction of PKD1 expression. The knockdown of PKD1 expression had no effect on cell proliferation because all three cell lines showed similar proliferation rates (Figure 3b). We next analysed if the knockdown of PKD1 had an impact on the invasiveness of the cells. Interestingly, cellular invasion in Matrigel Transwell assays was increased three to four-fold when PKD1 was knocked down (Figure 3c). Finally, we analysed the invasive potential of control and PKD1-shRNA MCF-7 cells in 3D cell culture. Cells were embedded in Matrigel and invasive growth was monitored over a period of 60 days. We observed that the multicellular MCF-7 spheroids showed a more invasive phenotype when PKD1 expression was reduced (Figure 3d). These results clearly indicate that the loss of PKD1 in very low-invasive tumour cells increases their invasive potential in two-dimensional (2D) and 3D culture systems.

### Active PKD1 inhibits breast tumour cell invasion

**Figure 4:**
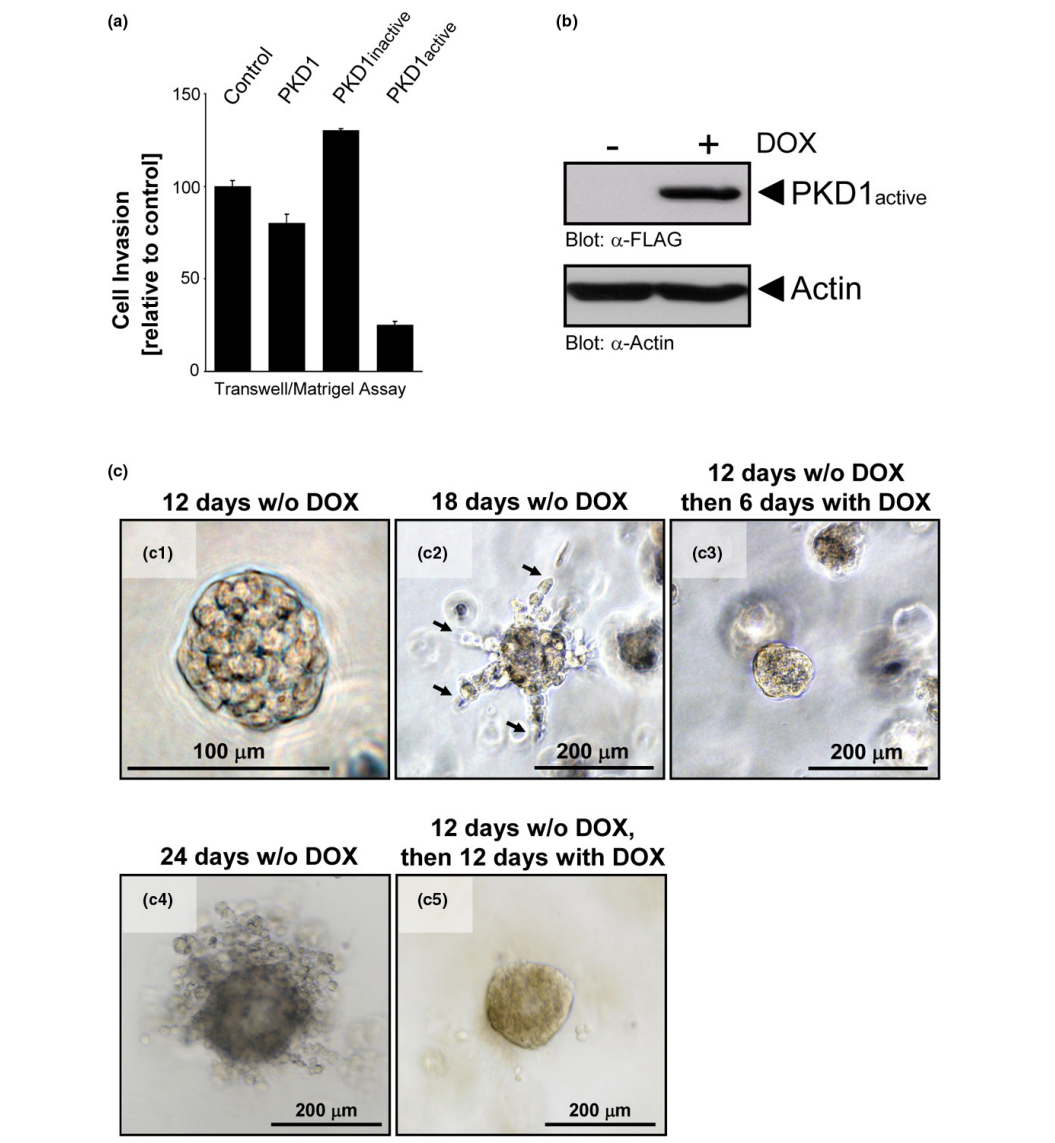
Active PKD1 inhibits breast tumour cell invasion. **(a) **MDA-MB-231 cells were transiently transfected with wildtype protein kinase D (PKD) 1, kinase-dead PKD1 (PKD1inactive) or constitutively-active PKD1 (PKD1active). After 24 hours cells were seeded on Matrigel-coated Transwell filters and Transwell invasion assays were performed over a time period of 16 hours. **(b) **Inducible expression of active PKD1 in MDA-MB-231-TR-PKD1active (PKD1.Y463E mutant) cells. Cells were treated with doxycyclin (DOX) for 16 hours. Cells were lysed and lysates were analysed by western blotting for expression of constitutively-active PKD1 (anti-FLAG) or actin (loading control). **(c)** MDA-MB-231-TR-PKD1.Y463E cells were seeded in 3D culture and were either left untreated for 12 (c1), 18 (c2) and 24 days (c4), or were treated with doxycyclin after 12 days of normal growth to induce the expression of active PKD1 (c3 and c5). Cells were photographed at day 12 (c1), day 18 (c2 and c3) or day 24 (c4 and c5). Arrows in c2 indicate cells invading from the spheroid into the extracellular matrix. Bars indicate the size of the spheroids.

We next determined if the re-expression of constitutively-active PKD1 impairs the invasive phenotype of the highly-invasive MDA-MB-231 cells. First, we transiently-transfected MDA-MB-231 cells with wildtype, constitutively-active (PKD1active, PKD1.Y463E mutant) or kinase-inactive (PKD1inactive, PKD1.K610W mutant) PKD1 alleles and measured their invasiveness in Matrigel Transwell assays. We found that the expression of constitutively-active PKD1 significantly inhibited cell invasion through Matrigel (Figure 4a). Wildtype PKD1 moderately decreased and kinase-inactive PKD1 slightly increased cell invasiveness (Figure 4a). To test long-term effects of expression of active PKD1 on cell invasion in the same cell line, we then generated a MDA-MB-231 cell line that allowed inducible expression of a constitutively-active PKD1 via doxycyclin. Doxycyclin induced the expression of constitutively-active PKD1 (PKD1active) within 24 hours (Figure 4b) and we did not see any leakage of this system. In Matrigel Transwell assays, the induction of constitutively-active PKD1 inhibited tumour cell invasion in a similar way shown for cells transiently-transfected with active PKD1 (data not shown).

We then utilised this inducible system to determine if active PKD1 affects the invasive behaviour of MDA-MB-231 cells growing in 3D cell culture. MDA-MB-231 cells growing in 3D culture in Matrigel within 12 days form multicellular spheroids with a size of approximately 80 μm (Figure 4c1). We found that from approximately day 18 this phenotype changes to a more stellate morphology, with projections of invasive cells emanating from a central multicellular spheroid (Figure 4c2). However, when spheroids were treated with doxycyclin at day 12 to induce the expression of active PKD1, the outgrowth of these invasive projections was blocked until day 18 (Figure 4c3). This indicates that the expression of PKD1 indeed blocks the invasive phenotype. The effect of PKD1 expression on cell invasiveness became even more apparent when cells were cultivated without doxycyclin for 24 days, where massive invasion from the spheroid into the surrounding ECM was observed (Figure 4c4). On the other hand, when cells were cultivated without doxycyclin for 12 days and then treated with doxycyclin to induce the expression of active PKD1 (12 days without and 12 days with doxycyclin), we observed significantly less cell invasion into the surrounding matrix (Figure 4c5). These results indicate that active PKD1 inhibits the invasion of breast cancer cells.

### Active PKD1 regulates the expression and activity of invasion-relevant MMPs

**Figure 5:**
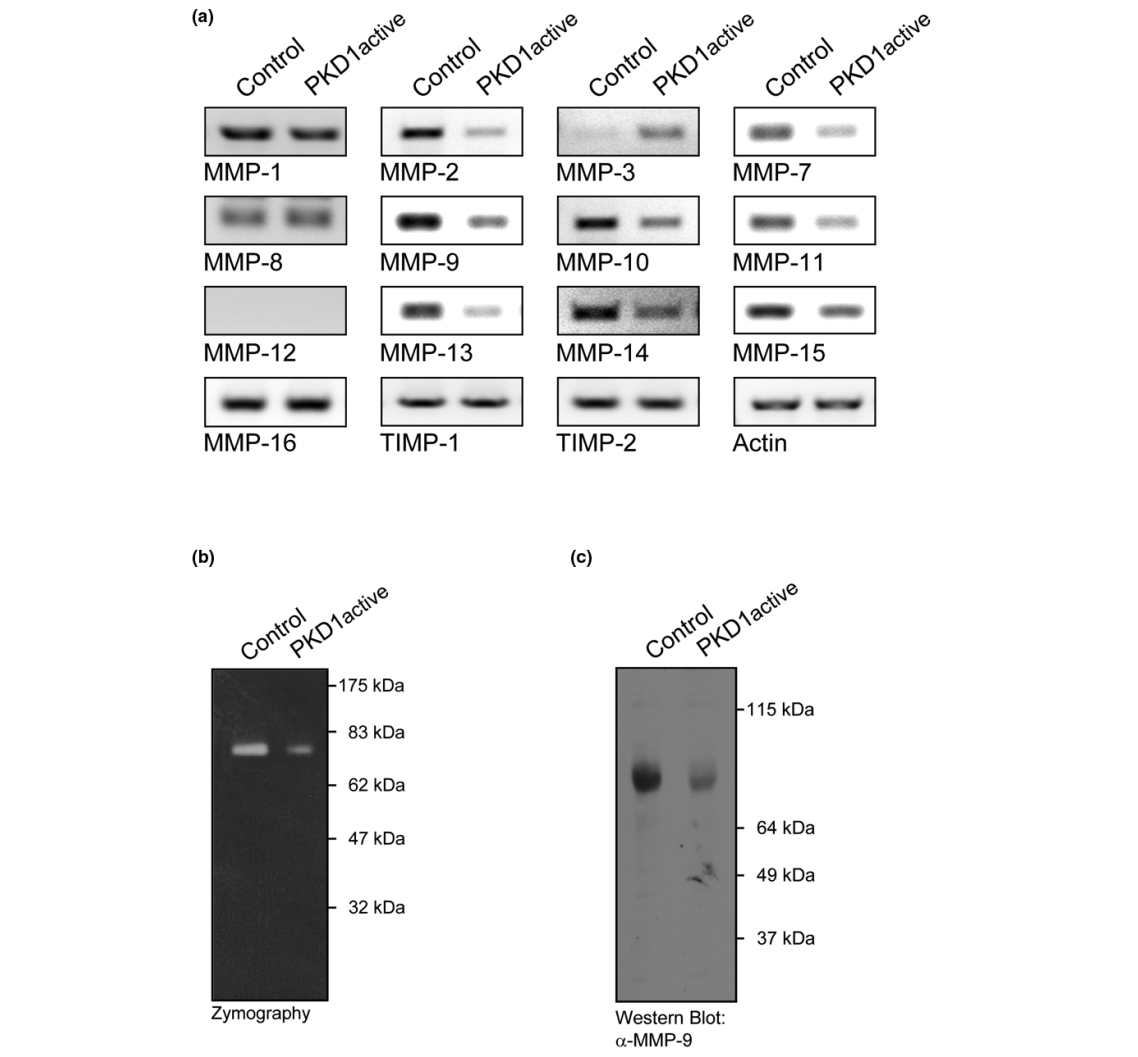
Active PKD1 regulates the expression of invasion-relevant MMPs. **(a) **MDA-MB-231 cells were transfected with vector control or constitutively-active protein kinase D (PKD) 1 (PKD1active, PKD1.Y463E mutant). After 16 hours mRNA was isolated and the expression of the matrix mettaloproteinases (MMP) MMP-1, MMP-2, MMP-3, MMP-7, MMP-8, MMP-9, MMP-10, MMP-11, MMP-12, MMP-13, MMP-14, MMP-15 and MMP-16, tissue inhibitors of metalloproteinases (TIMP) TIMP1 and TIMP2 and actin (control) was analysed by RT-PCR (shown as a PCR reaction with 35 cycles; PCR reaction with 20 and 40 cycles showed similar results). **(b,c)** MDA-MB-231 cells were transfected with vector control or constitutively-active PKD1 (PKD1active, PKD1.Y463E mutant). After 48 hours supernatants were collected and used for (b) zymographic analysis or (c) western blot analysis for MMP-9 expression.

It is known that breast tumour cells actively produce MMPs to facilitate tumour cell invasion. We therefore aimed to find out if PKD1 regulates the expression of a panel of MMPs. MDA-MB-231 cells are an established model for malignant human breast cancer cell invasion *in vitro*, and it has been shown that multiple MMPs, including MMP-1, MMP-2, MMP-7, MMP-9, MMP-11, MMP-12, MMP-13, MMP-14 and MMP-17, enhance cell invasiveness [37-39]. To test whether PKD1 impacts the expression of MMPs in MDA-MB-231 cells, we transfected them with constitutively-active PKD1 (PKD1active) and analysed the expression of multiple MMPs and TIMP. Using reverse transcription (RT) PCR, we analysed the expression of MMP-1, MMP-2, MMP-3, MMP-7, MMP-8, MMP-9, MMP-10, MMP-11, MMP12, MMP-13, MMP-14, MMP-15, MMP-16, TIMP1 and TIMP2, as well as actin which served as a loading control (Figure 5a). We found that the expression of constitutively-active PKD1 downregulated mRNA transcripts of MMP-2, MMP-7, MMP-9, MMP-10, MMP-11, MMP-13, MMP-14 and MMP-15. Of these MMPs, all but MMP-15 are known to enhance the invasiveness of MDA-MB-231 cells [37-39]. We did not observe differences in the expression of MMP-1, MMP-8, MMP-16, TIMP1, TIMP2 or actin. Further, the expression of MMP-3 was increased by active PKD1. This is interesting, because MMP-3 was previously shown to inhibit cell invasion of MDA-MB-231 [40].

We then performed gelatin zymographic analysis to test if the decreased expression of MMPs can relay to decreased MMP activity. Therefore, MDA-MB-231 cells were either transfected with vector control or with constitutively-active PKD1. We observed a significant decrease in MMP activity when constitutively-active PKD1 is expressed. This is most likely to be because of decreased MMP-2 (p72) and MMP-9 (p68) activity as the MMP activity was detected at a molecular weight of approximately 70 kDa (Figure 5b). Western blotting analysis for MMP-9 and MMP-2 showed that MMP-2 is not detectable in supernatants of MDA-MB-231 cells (data not shown), but that MMP-9 is decreased in cells expressing active PKD1 (Figure 5c). This suggests that MMP-9 is the mainly expressed MMP in MDA-MB-231 and that its expression is negatively-regulated by PKD1, which directly translates to decreased activity. Therefore, PKD1 mediates breast cancer cell invasion through regulation of the expression of invasion-relevant MMPs.

## Discussion

To develop effective therapies that stop breast cancer from metastasising, the underlying biological and molecular events need to be understood in further detail. We show here that the PKD family members PKD1, PKD2 and PKD3 are all expressed in ductal epithelial cells of the normal breast (Figure 1). We further show that decreased expression of PKD1 can serve as a marker for invasive breast cancer, whereas PKD2 and PKD3 expression remain unchanged in normal breast and invasive breast tumour tissue (Figure 1). However, all three PKD enzymes are markers for breast epithelial cells (normal and tumour) and may be utilised as markers to identify breast epithelia-derived metastases. PKD1 expression was downregulated by approximately 60% in more than 95% of the analysed samples of invasive ductal carcinoma and distant lymph node metastases (Figure 1b). All 50 analysed tumours were assessed by pathologists and stages were at a range from 0, IIA, IIB, IIIA, IIIB, IIIC. Further, additional information such as sex, age, diagnosis, pTNM, lymph node stage (positive lymph nodes/examined lymph nodes), as well as expression of the PR, or the more-aggressive ER-negative, basal sub-type of breast cancer were available. Downregulation of PKD1 expression occurred in more than 95% of the analysed cases of invasive ductal cancer and no correlation was observed with stage, ER, PR or other markers. Our results on PKD1 in invasive breast cancer are in consensus with data obtained for gastric cancer and prostate cancer, where decreased expression of PKD1 was described in most of the cases analysed [14,15].

Our data showing reduced PKD1 protein expression in invasive breast cancer is also in consensus with published transcriptional microarray data profiling over 350 surgically excised, advanced breast tumour tissues. In these arrays *PRKD1 *gene expression was drastically reduced in most cases analysed [41-44]. Our data show that reduced gene expression invariably translates to decreased protein levels. Investigation of other publicly available microarray datasets on the NCBI Gene Expression Omnibus (GEO) showed that *PRKD1 *is detected at appreciable levels in normal lobular and ductal breast cells [GEO:GDS2635] [45], in atypical hyperplasia [GEO:GDS1250] [46] and in the cancerous lesions invasive ductal and lobular carcinomas [GEO:GDS2635] [45], suggesting that PKD1 expression is indeed decreased with increased invasiveness of the tumours.

Little is known about the role of PKD1 in regulating tumour cell migration and invasion, important processes that regulate both tumour expansion and metastasis. In order to investigate a potential role for PKD1 in cell invasion, we first compared PKD1 expression in very low-invasive and highly invasive breast cancer cell lines (Figures 2a,c) and found that from the three PKD family members only PKD1 showed a significant expression pattern associated with the invasive phenotype. PKD1 expression was absent in highly invasive breast cancer cell lines including MDA-MB-231, T47D and SKBR3 (Figure 2). This is most likely because of epigenetic silencing mediated by DNA methyltransferases (Figure 2d). Non-invasive or very low-invasive breast cancer cell lines such as BT-474 or MCF-7 and the normal breast cancer cell line MCF-10A moderately expressed PKD1. Moreover, by analysing PKD1 expression in the 1-HMT-3522 breast cancer cell progression model, we found that the T4/2 clone which shows increased invasiveness as compared with the S1 clone also expressed less PKD1 (Figure 2b).

We utilised two breast cancer model cell lines, MCF-7 and MDA-MB-231, to investigate the role of PKD1 in cell invasion. MCF-7 and MDA-MB-231 cells express comparable amounts of PKD2 and PKD3, but differ in their expression of PKD1 (Figure 2). The depletion of PKD1 in MCF-7 cells resulted in increased cell invasion in both 2D and 3D cell culture systems (Figure 3).

On the other hand, the re-introduction of active PKD-1 in MDA-MB-231 cells impaired their invasive behaviour in 2D and 3D cell culture (Figure 4). Notably, the knockdown of PKD1 in MCF-7 cells (Figure 3B) and the induction of constitutively active PKD1 in MDA-MB-231 cells had no significant effects on cell proliferation or cell death (data not shown). This is interesting, because one of the PKD family members, PKD3, was recently linked to increased tumour cell proliferation in prostate cancer [47]. This implies that in different cancers the three PKD family members may have different functions. A similar phenomenon was recently demonstrated for the kinase Akt, which, depending on the isoenzyme expressed, contributes to breast tumour cell survival and proliferation, or blocks cell migration and invasion [48]. Cell proliferation, survival and cell motility are not necessarily linked in cancer cells, and it is generally accepted in the field that proliferation and invasiveness are independent of each other.

Our data further suggest that PKD1 inhibits breast cancer cell invasion by regulating the expression of factors involved in the degradation of ECM. The invasion of MDA-MB-231 cells in Matrigel is dependent on MMPs. For example, MMP-2, MMP-7, MMP-9, MMP-11, MMP-13 and MMP-14 are known to enhance the invasiveness of MDA-MB-231 cells [37-39]. We found that the expression of active PKD1 in MDA-MB-231 cells downregulated mRNA transcripts of MMP-2, MMP-7, MMP-9, MMP-10, MMP-11, MMP-13, MMP-14 and MMP-15 (Figure 5a). Thus, PKD1 decreased the expression of all MMPs so far implicated in the invasive phenotype of this cell line. The mechanism of how PKD1 regulates such a multitude of genes is not known yet. One explanation is that PKD1 may regulate a common element in the promoter of these MMPs. In this context histone deacetylases (HDACs) have been shown to regulate the expression of MMPs [30,31]. PKD1 is known to be a negative regulator of HDACs [49] and it is possible that PKD1 exerts its effects on all the MMPs via regulation of HDACs.

We did not observe any differences in the expression of MMP-1, MMP-8, MMP-16, TIMP1 or TIMP2 by PKD1. Further, the expression of MMP-3 was slightly increased by active PKD1. This is interesting, because MMP-3 has been previously shown to inhibit cell invasion of MDA-MB-231 [40]. MMP-3 expression was associated with benign and early stage breast tumours but is frequently lost in advanced stage, aggressive breast disease [40]. The events leading to the transition from a benign to a metastatic tumour are not fully understood, but are linked to ECM degradation and increased motility of cells. It is possible that loss of PKD1 expression and the resulting change in the expression of MMPs is part of the switch driving the progression from a benign to an invasive, malignant tumour.

## Conclusions

Our results show that decreased PKD1 expression can serve as a marker for invasive and metastatic breast cancer. They further suggest that the loss of PKD1 expression increases the malignant potential of breast cancer cells. This may be because of the function of PKD1 as a negative regulator of MMP expression. This knowledge can be applied to develop new therapeutic avenues such as the re-expression of PKD1 as one potential strategy to ameliorate breast cancer metastasis.

## Abbreviations

2D: two-dimensional; 3D: three-dimensional; ATCC: American Type Culture Collection; BSA: bovine serum albumin; ECM: extracellular matrix; ER: oestrogen receptor; HDAC: histone deacetylase; HRP: horseradish peroxidase; MMP: matrix-metalloproteinase; MT: membrane type; NaCl: sodium chloride; NF-κB: nuclear factor-κB; PBS: phosphate buffered saline; PKC: protein kinase C; PKD: protein kinase D; PR: progesterone receptor; pTNM: pathological tumour-node-metastasis; RT-PCR: reverse transcription polymerase chain reaction; TIMP: tissue inhibitors of metalloproteinases; TMA: tissue microarray.

## Competing interests

The authors declare that they have no competing interests.

## Authors' contributions

TE, HD and IY performed the experiments. PS was responsible for the study concept and design and wrote the manuscript. SG contributed to writing the manuscript and performed microarray data analysis.

## References

[B1] Storz P, Doppler H, Toker A (2005). Protein kinase D mediates mitochondrion-to-nucleus signaling and detoxification from mitochondrial reactive oxygen species. Mol Cell Biol.

[B2] Rykx A, De Kimpe L, Mikhalap S, Vantus T, Seufferlein T, Vandenheede JR, Van Lint J (2003). Protein kinase D: a family affair. FEBS letters.

[B3] Hausser A, Storz P, Martens S, Link G, Toker A, Pfizenmaier K (2005). Protein kinase D regulates vesicular transport by phosphorylating and activating phosphatidylinositol-4 kinase IIIbeta at the Golgi complex. Nat Cell Biol.

[B4] Bowden ET, Barth M, Thomas D, Glazer RI, Mueller SC (1999). An invasion-related complex of cortactin, paxillin and PKCmu associates with invadopodia at sites of extracellular matrix degradation. Oncogene.

[B5] Endo K, Oki E, Biedermann V, Kojima H, Yoshida K, Johannes FJ, Kufe D, Datta R (2000). Proteolytic cleavage and activation of protein kinase C μ by caspase-3 in the apoptotic response of cells to 1-beta-D-arabinofuranosylcytosine and other genotoxic agents. J Biol Chem.

[B6] Palmantier R, Roberts JD, Glasgow WC, Eling T, Olden K (1996). Regulation of the adhesion of a human breast carcinoma cell line to type IV collagen and vitronectin: roles for lipoxygenase and protein kinase C. Cancer Res.

[B7] Rozengurt E, Rey O, Waldron RT (2005). Protein kinase D signaling. J Biol Chem.

[B8] Storz P, Toker A (2003). Protein kinase D mediates a stress-induced NF-kappaB activation and survival pathway. EMBO journal.

[B9] Storz P (2005). Reactive oxygen species in tumor progression. Front Biosci.

[B10] Eiseler T, Schmid MA, Topbas F, Pfizenmaier K, Hausser A (2007). PKD is recruited to sites of actin remodelling at the leading edge and negatively regulates cell migration. FEBS letters.

[B11] Song J, Li J, Lulla A, Evers BM, Chung DH (2006). Protein kinase D protects against oxidative stress-induced intestinal epithelial cell injury via Rho/ROK/PKC-δ pathway activation. Am J Physiol Cell Physiol.

[B12] Simpson KJ, Dugan AS, Mercurio AM (2004). Functional analysis of the contribution of RhoA and RhoC GTPases to invasive breast carcinoma. Cancer Res.

[B13] Wang W, Eddy R, Condeelis J (2007). The cofilin pathway in breast cancer invasion and metastasis. Nat Rev Cancer.

[B14] Jaggi M, Rao PS, Smith DJ, Hemstreet GP, Balaji KC (2003). Protein kinase C mu is down-regulated in androgen-independent prostate cancer. Biochem Biophys Res Commun.

[B15] Kim M, Jang HR, Kim JH, Noh SM, Song KS, Cho JS, Jeong HY, Norman JC, Caswell PT, Kang GH, Kim SY, Yoo HS, Kim YS (2008). Epigenetic inactivation of protein kinase D1 in gastric cancer and its role in gastric cancer cell migration and invasion. Carcinogenesis.

[B16] Egeblad M, Werb Z (2002). New functions for the matrix metalloproteinases in cancer progression. Nat Rev Cancer.

[B17] Sternlicht MD, Lochter A, Sympson CJ, Huey B, Rougier JP, Gray JW, Pinkel D, Bissell MJ, Werb Z (1999). The stromal proteinase MMP3/stromelysin-1 promotes mammary carcinogenesis. Cell.

[B18] Coussens LM, Fingleton B, Matrisian LM (2002). Matrix metalloproteinase inhibitors and cancer: trials and tribulations. Science.

[B19] Chabottaux V, Noel A (2007). Matrix metalloproteinases to predict breast cancer metastases. Clin Lab Int.

[B20] Duffy MJ, Maguire TM, Hill A, McDermott E, O'Higgins N (2000). Metalloproteinases: role in breast carcinogenesis, invasion and metastasis. Breast Cancer Res.

[B21] Martin M, Matrisian L (2004). Matrix metalloproteinases as prognostic factors for cancer. Clin Lab Int.

[B22] Zhang B, Cao X, Liu Y, Cao W, Zhang F, Zhang S, Li H, Ning L, Fu L, Niu Y, Niu R, Sun B, Hao X (2008). Tumor-derived matrix metalloproteinase-13 (MMP-13) correlates with poor prognoses of invasive breast cancer. BMC Cancer.

[B23] Matrisian LM (1990). Metalloproteinases and their inhibitors in matrix remodeling. Trends Genet.

[B24] Turpeenniemi-Hujanen T (2005). Gelatinases (MMP-2 and -9) and their natural inhibitors as prognostic indicators in solid cancers. Biochimie.

[B25] Minn AJ, Gupta GP, Siegel PM, Bos PD, Shu W, Giri DD, Viale A, Olshen AB, Gerald WL, Massague J (2005). Genes that mediate breast cancer metastasis to lung. Nature.

[B26] Gupta GP, Nguyen DX, Chiang AC, Bos PD, Kim JY, Nadal C, Gomis RR, Manova-Todorova K, Massague J (2007). Mediators of vascular remodelling co-opted for sequential steps in lung metastasis. Nature.

[B27] Nelson KK, Melendez JA (2004). Mitochondrial redox control of matrix metalloproteinases. Free Radic Biol Med.

[B28] Thomas P, Khokha R, Shepherd FA, Feld R, Tsao MS (2000). Differential expression of matrix metalloproteinases and their inhibitors in non-small cell lung cancer. J Pathol.

[B29] Westermarck J, Kahari VM (1999). Regulation of matrix metalloproteinase expression in tumor invasion. Faseb J.

[B30] Liu LT, Chang HC, Chiang LC, Hung WC (2003). Histone deacetylase inhibitor up-regulates RECK to inhibit MMP-2 activation and cancer cell invasion. Cancer Res.

[B31] Klampfer L, Huang J, Shirasawa S, Sasazuki T, Augenlicht L (2007). Histone deacetylase inhibitors induce cell death selectively in cells that harbor activated kRasV12: The role of signal transducers and activators of transcription 1 and p21. Cancer Res.

[B32] Bartsch JE, Staren ED, Appert HE (2003). Matrix metalloproteinase expression in breast cancer. J Surg Res.

[B33] Jauliac S, Lopez-Rodriguez C, Shaw LM, Brown LF, Rao A, Toker A (2002). The role of NFAT transcription factors in integrin-mediated carcinoma invasion. Nature Cell Biol.

[B34] Leber TM, Balkwill FR (1997). Zymography: a single-step staining method for quantitation of proteolytic activity on substrate gels. Anal Biochem.

[B35] Storz P (2007). Mitochondrial ROS – radical detoxification, mediated by protein kinase D. Trends Cell Biol.

[B36] Wang F, Hansen RK, Radisky D, Yoneda T, Barcellos-Hoff MH, Petersen OW, Turley EA, Bissell MJ (2002). Phenotypic reversion or death of cancer cells by altering signaling pathways in three-dimensional contexts. J Natl Cancer Inst.

[B37] Hegedus L, Cho H, Xie X, Eliceiri GL (2008). Additional MDA-MB-231 breast cancer cell matrix metalloproteinases promote invasiveness. J Cell Physiol.

[B38] Hotary K, Li XY, Allen E, Stevens SL, Weiss SJ (2006). A cancer cell metalloprotease triad regulates the basement membrane transmigration program. Genes Dev.

[B39] Ramos-DeSimone N, Hahn-Dantona E, Sipley J, Nagase H, French DL, Quigley JP (1999). Activation of matrix metalloproteinase-9 (MMP-9) via a converging plasmin/stromelysin-1 cascade enhances tumor cell invasion. J Biol Chem.

[B40] Farina AR, Tacconelli A, Cappabianca L, Gulino A, Mackay AR (2002). Inhibition of human MDA-MB-231 breast cancer cell invasion by matrix metalloproteinase 3 involves degradation of plasminogen. Eur J Biochem.

[B41] Farmer P, Bonnefoi H, Becette V, Tubiana-Hulin M, Fumoleau P, Larsimont D, Macgrogan G, Bergh J, Cameron D, Goldstein D, Duss S, Nicoulaz AL, Brisken C, Fiche M, Delorenzi M, Iggo R (2005). Identification of molecular apocrine breast tumours by microarray analysis. Oncogene.

[B42] Perou CM, Sorlie T, Eisen MB, Rijn M van de, Jeffrey SS, Rees CA, Pollack JR, Ross DT, Johnsen H, Akslen LA, Fluge O, Pergamenschikov A, Williams C, Zhu SX, Lønning PE, Børresen-Dale AL, Brown PO, Botstein D (2000). Molecular portraits of human breast tumours. Nature.

[B43] Vijver MJ van de, He YD, van't Veer LJ, Dai H, Hart AA, Voskuil DW, Schreiber GJ, Peterse JL, Roberts C, Marton MJ, Parrish M, Atsma D, Witteveen A, Glas A, Delahaye L, Velde T van der, Bartelink H, Rodenhuis S, Rutgers ET, Friend SH, Bernards R (2002). A gene-expression signature as a predictor of survival in breast cancer. N Engl J Med.

[B44] van 't Veer LJ, Dai H, Vijver MJ van de, He YD, Hart AA, Mao M, Peterse HL, Kooy K van der, Marton MJ, Witteveen AT, Schreiber GJ, Kerkhoven RM, Roberts C, Linsley PS, Bernards R, Friend SH (2002). Gene expression profiling predicts clinical outcome of breast cancer. Nature.

[B45] Turashvili G, Bouchal J, Baumforth K, Wei W, Dziechciarkova M, Ehrmann J, Klein J, Fridman E, Skarda J, Srovnal J, Hajduch M, Murray P, Kolar Z (2007). Novel markers for differentiation of lobular and ductal invasive breast carcinomas by laser microdissection and microarray analysis. BMC Cancer.

[B46] Poola I, DeWitty RL, Marshalleck JJ, Bhatnagar R, Abraham J, Leffall LD (2005). Identification of MMP-1 as a putative breast cancer predictive marker by global gene expression analysis. Nat Med.

[B47] Chen J, Deng F, Singh SV, Wang QJ (2008). Protein kinase D3 (PKD3) contributes to prostate cancer cell growth and survival through a PKCepsilon/PKD3 pathway downstream of Akt and ERK 1/2. Cancer Res.

[B48] Yoeli-Lerner M, Yiu GK, Rabinovitz I, Erhardt P, Jauliac S, Toker A (2005). Akt blocks breast cancer cell motility and invasion through the transcription factor NFAT. Mol Cell.

[B49] Ha CH, Wang W, Jhun BS, Wong C, Hausser A, Pfizenmaier K, McKinsey TA, Olson EN, Jin ZG (2008). Protein kinase D-dependent phosphorylation and nuclear export of histone deacetylase 5 mediates vascular endothelial growth factor-induced gene expression and angiogenesis. J Biol Chem.

